# Anti-arthritic and endothelial protective effects of *Derris scandens* extract in adjuvant-induced arthritis in rats

**DOI:** 10.1371/journal.pone.0337472

**Published:** 2025-12-11

**Authors:** Nattiya Chaichamnong, Corine Girard, Maude Tournier, Francis Bonnefoy, Kornkanok Ingkaninan, Céline Demougeot, Perle Totoson

**Affiliations:** 1 Université Marie et Louis Pasteur, EFS, INSERM UMR1098 RIGHT, Besançon, France; 2 Center of Excellence for Natural Health Product Innovation and Center of Excellence for Innovation in Chemistry, Faculty of Pharmaceutical Sciences, Naresuan University, Phitsanulok, Thailand; University of Illinois, UNITED STATES OF AMERICA

## Abstract

Rheumatoid arthritis is a chronic inflammatory disease associated with a significantly increased risk of cardiovascular mortality. This study investigated the therapeutic potential of *Derris scandens* (Roxb.) Benth. stem extract (DS) in mitigating vascular and cardiac damage associated with arthritis in an adjuvant-induced arthritis (AIA) rat model. Treatment with DS (200 mg/kg/day, p.o.), methotrexate (1 mg/kg/week, s.c.), or their combination was administered post-induction from day 11–32. Body weight, arthritis scores, and paw diameters were monitored daily. At the end of the treatment period, electrocardiographic parameters and blood pressure were assessed in anesthetized rats. Endothelial function was evaluated in isolated pulmonary arteries (PA) and aortae via acetylcholine-induced relaxation. Plasma inflammatory cytokines were quantified, and leukocyte populations were analyzed in blood and PA tissues. The direct vascular effects of DS were also examined in healthy rats. Direct exposure to DS induced greater relaxation in the PA (E_max_ = 90%) than in the aorta (E_max_ = 38%). When administered to arthritic rats, DS significantly reduced arthritis scores and paw diameters compared to untreated rats. Furthermore, DS markedly improved endothelial function in both the PA and aorta, in conjunction with reductions in plasma levels of TNF-α and IL-1β, as well as a decrease in leukocyte populations, particularly neutrophils. However, co-administration with methotrexate attenuated the vascular effects. No significant changes were observed in body weight, electrocardiographic parameters, or blood pressure across treatment groups. This study highlights the potential of DS in improving endothelial function and in mitigating arthritis-related inflammation. Our data also supports the role of vascular neutrophil infiltration in the manifestation of endothelial dysfunction.

## Introduction

Rheumatoid arthritis (RA) is a chronic inflammatory disease characterized by microvascular injury, an increase in synovial lining cells, such as macrophages and neutrophils, lymphocyte infiltration within the synovium, and elevated levels of various inflammatory cytokines [1]. It is well established that RA is associated with increased cardiovascular mortality, due to atherosclerosis, endothelial dysfunction (ED), and cardiac disorders [2,3]. Although current treatments can achieve remission in 43–81% of patients [4], cardiovascular risk remains elevated [5]. Disease-modifying antirheumatic drugs (DMARDs) do not equally improve endothelial function or mitigate cardiac disorders [6]. For instance, methotrexate (MTX), the first-line treatment for RA, does not reduce ED [7,8], despite a general reduction in the cardiovascular risk of patients with RA [9]. This highlights the potential value of adjunct therapies, such as medicinal plants, in reducing cardiovascular risks in patients with RA.

The adjuvant-induced arthritis (AIA) rat model is widely used to unravel the RA mechanisms due to its ability to replicate clinical characteristics observed in patients. Commonly induced through a single subcutaneous injection of heat-killed *Mycobacterium butyricum* suspended in Freund’s Adjuvants, this model triggers robust inflammatory responses mediated by T-cells and neutrophils, leading to increased expression of cytokines such as tumor necrosis factor (TNF)-α, interleukin (IL)-1β, IL-6, and IL-17 [10,11]. The inflammatory cascade in the AIA model not only contributes to joint damage but also induces ED and cardiovascular complications [12]. Previous studies have demonstrated that medicinal plant extracts and natural compounds with anti-inflammatory properties can effectively modulate arthritis progression in the AIA model [13,14]. Nevertheless, to the best of our knowledge, studies examining the impact of medicinal plants on the cardiovascular consequences of arthritis remain scarce.

*Derris scandens* (Roxb.) Benth. is a synonym for *Brachypterum scandens* (Roxb.) Wight & Arn. ex Miq. and belongs to the Fabaceae family. In this study, we adopt the name *Derris scandens*, which is more widely accepted and frequently used in recent literature. Traditionally, its stem has been used orally to treat musculoskeletal and joint pain, inflammatory conditions, and dysentery [15,16]. The traditional uses of *D. scandens* for treating musculoskeletal and joint pain have been clinically validated [17,18]. The pharmacological properties of *D. scandens* have been characterized through animal models of skin and subplantar inflammation, as well as *in vitro* studies. Laupattarakasem and colleagues demonstrated that an aqueous stem extract of *D. scandens* exhibited potent anti-inflammatory effects by significantly reducing myeloperoxidase release, an enzyme associated with tissue damage in arthritis. Furthermore, that study found that the extract, administered intraperitoneally, significantly reduced carrageenan-induced paw edema [19]. The mechanisms underlying the anti-inflammatory effects of *D. scandens* stem extract have been partially elucidated and included the inhibition of NO production and the downregulation of pro-inflammatory cytokine gene expression, such as COX-2 [20], COX-1 [21], IL-1α, IL-1β, IL-6, inducible nitric oxide synthase (iNOS), matrix metalloproteinases (MMP-1 and MMP-9) [20], and lipoxygenase (LOX) [19]. Additionally, a reduction in eicosanoid production has been observed [21]. However, data from relevant animal models of systemic arthritis remain limited.

Emerging evidence from experimental models suggests that *D. scandens* extract exhibits cardiovascular activities linked to its phosphodiesterase-5 (PDE5) inhibition [22], which may contribute to improving endothelial function. Additionally, *D. scandens* extract was shown to have β-adrenergic receptor antagonism [21], prompting us to evaluate its cardiovascular effects in the case of an arthritis condition.

In our study, our purpose was to determine whether DS treatment, alone or in combination with methotrexate (MTX), could improve endothelial function and mitigate associated cardiac disorders in this model. We also explored the underlying mechanisms of the observed effects. Initially, we assessed the impact of the treatments on endothelial function in the pulmonary artery (PA), which exhibits high PDE5 expression, and the aorta, which serves systemic circulation. Blood pressure and electrocardiogram (ECG) parameters were measured post-treatment. Additionally, in healthy rats, the direct vasorelaxant effect of DS was evaluated *ex vivo* in isolated aortae and pulmonary arteries. This approach is expected to enhance traditional knowledge of *D. scandens* and highlight its potential as a supportive therapy in the overall management of arthritis.

## Materials and methods

### Reagents

N_ω_-nitro-L-arginine methyl ester (L-NAME), acetylcholine (ACh), apamin, indomethacin, and phenylephrine (PE) were purchased from Sigma‐Aldrich (St. Louis, MO, USA). Charybdotoxin was obtained from AnaSpec (Fremont, CA, USA). Sodium pentobarbital was obtained from Ceva Santé Animale, France. All chemical reagents were of pharmaceutical grade.

### Plant material and extraction

Dried *D. scandens* stem material was purchased in March 2022 from Charoensuk Pharma Supply Co., Ltd., Nakhon Pathom, Thailand. The plant specimen was authenticated by the Division of Applied Thai Traditional Medicine, Faculty of Public Health, Naresuan University, Thailand, and voucher specimen No. 004331 was deposited in the PNU Herbarium, Faculty of Science, Naresuan University, Phitsanulok, Thailand. The dry plant material (1 kg) was ground into a powder and macerated with 95% ethanol (4 L) for 24 h, three times. The filtrate was combined and concentrated using a rotary evaporator under reduced pressure at 30°C to 35°C. The *D. scandens* stem extract (DS) was a viscous brown substance with a yield of 3.65%.

The chemical composition of DS was characterized using our published validated LC-QTOF-MS/MS method [22], revealing 3.49% w/w of lupalbigenin.

### Animals

Seventy-eight male Lewis (6-week-old) and eight male Wistar rats (8-week-old) were obtained from JanvierLabs (Le Genest Saint Isle, France). They were housed in cages, 2–3 per cage, under a 12-h light-dark cycle, with the temperature maintained at 22 ± 1°C, and supplied with standard food and water *ad libitum*. The experimental protocols involving rats No. 2019–003-PT-5PR were approved by the Ethics Committees of the Université Marie et Louis Pasteur (Besançon, France) and adhered to the ARRIVE (Animal Research: Reporting In Vivo Experiments) guidelines. The euthanasia method was selected for terminal procedures requiring tissue collection and is consistent with the AVMA’s recommendations for non-recovery euthanasia in laboratory animals.

### Vascular reactivity protocol

To determine if DS directly affects endothelial function, the impact of the cumulative concentration of DS on arteries from the systemic circulation (aorta) and the pulmonary circulation (pulmonary artery, PA) was explored in healthy Wistar rats. The rats were sedated with sodium pentobarbital (60 mg/kg, i.p.), and euthanasia was performed via exsanguination of the abdominal aorta. Then, the thoracic aorta and PA were excised, cleaned of connective tissue, and cut into rings of approximately 2 mm in length. Rings were suspended in Krebs solution (NaCl 118 M, KCl 4.65 M, CaCl_2_ 2.5 M, KH_2_PO_4_ 1.18 M, NaHCO_3_ 24.9 M, MgSO_4_ 1.18 M, glucose 12 M, pH 7.4), maintained at 37°C and continuously aerated with 95% O_2_, 5% CO_2_, for isometric tension recording in an organ chamber, as previously described [23,24]. In some rings, the endothelium was mechanically removed. The completeness of endothelial denudation was confirmed by the absence of relaxation to the endothelium-dependent agonist acetylcholine (ACh, 10^−6^ M for aorta and 10^−5^ M for PA). After a 90-min equilibration period under a resting tension of 2 g (aorta) or 1 g (PA), endothelium-intact and endothelium-denuded rings were constricted with phenylephrine (PE, 10^−6^ M for aorta and 10^−5^ M for PA) and exposed to a cumulative concentration of DS (0.01 to 300 µg/mL). Dimethyl sulfoxide (DMSO) was used as a vehicle. To evaluate the roles of NOS (nitric oxide synthase), COX-2 (cyclooxygenase-2), and EDHFs (endothelium-derived hyperpolarizing factors), the rings were pre-incubated for 1 hr with the following inhibitors: a non-selective NOS inhibitor, L-NAME (10^−4^ M); a COX inhibitor, indomethacin (10^−5^ M), and Ca^2+^-dependent K^+^ channels inhibitors, apamin (10^−7^ M) and charybdotoxin (10^−7^ M). The relaxation was calculated and expressed as a percentage relaxation in response to PE-induced contraction.

### Adjuvant-induced arthritis (AIA) model

The anti-arthritic inflammatory effects of DS were evaluated using the AIA rat model. Arthritis was induced in 6-week-old Lewis male rats by a single subcutaneous injection of 120 µL of 10 mg of heat-killed *Mycobacterium butyricum* suspended in 1 mL of Freund’s incomplete adjuvant at the base of the tail, as described previously [12].

### Assessment of arthritis

The rats were weighed daily, measured for hind paw diameters, and visually examined for clinical signs of arthritis. The arthritis scores consisted of a finger score of 0.1, weak and moderate arthritis of a big joint (ankle or wrist) score of 0.5, and intense arthritis of a big joint score of 1. The tarsus and ankle were considered as one joint. The sum of the joint scores of four limbs led to a maximum arthritis score of 6 for each rat [25].

### Experimental design and drug administration

Experiments were conducted on two series for the investigation of endothelial function (“endothelial function” series) and leukocyte count in PA and blood (“leukocyte count” series).

On the day of the first inflammatory symptoms (i.e., day 11–12 post-immunization), AIA rats from each series were randomized into five treatment groups and treated for 21 days. One group received DS at 200 mg/kg (p.o.) daily (« AIA-DS »), another group received MTX at 1 mg/kg (s.c.) once a week (« AIA-MTX »), a third group received DS at 200 mg/kg (p.o.) daily plus MTX at 1 mg/kg (s.c.) once a week (« AIA-MTXD »), a disease control group received the vehicles, 1 mL/kg propylene glycol (p.o.) and 1 mL/kg PBS (s.c.) once a week (« AIA-vehicle »). The final group was normal non-arthritic rats (« Normal control »). The rat numbers in each serial were as follows;

- “Endothelial function” series: AIA-DS (n = 13), AIA-MTX (n = 11), AIA-MTXD (n = 14), AIA-vehicle (n = 10)
- “Leukocyte count” series: AIA-DS, AIA-MTX, AIA-MTXD, AIA-vehicle, Normal control (n = 6, each)

The dosage of MTX was chosen based on prior data from the AIA model, which demonstrated a significant reduction in arthritis severity [7]. The dosage of DS (200 mg/kg) was selected based on our preliminary study, which assessed its efficacy in reducing inflammation in AIA rats.

### Tissue collection and electrocardiogram (ECG) measurements

Tissue collection and all data acquisition were performed blindly. Twenty-one days after treatment initiation, the rats were sedated by intraperitoneal injection of sodium pentobarbital (60 mg/kg, i.p.). The depth of sedation and unconsciousness was confirmed by the absence of reflex responses, such as the pedal withdrawal reflex. ECG analysis was performed, and blood pressure (BP) measurements, including systolic blood pressure (SBP), diastolic blood pressure (DBP), and mean arterial pressure (MAP), were recorded via carotid cannulation of the left carotid artery, utilizing LabChart Ver. 7.3.4 software (ADInstruments, Paris, France). Following these procedures, euthanasia was performed by exsanguination via the abdominal aorta.

In the “endothelial function” series, collected blood was centrifuged to obtain plasma, divided into aliquots, and stored at −80°C until analysis for inflammatory cytokine levels. After cardiac exsanguination, the thoracic aorta and PA were removed and immediately used for the vascular reactivity study. In the “leukocyte count” series, whole blood was collected. One milliliter was transferred into a K2E Microtainer (BD Biosciences, Le Pont-de-Claix, France). The PA was carefully removed, rinsed in cold saline solution, weighed, and immediately processed for flow cytometry analysis.

### Flow cytometry analysis

The flow cytometry protocol was performed as previously described [26,27]. PA tissues were digested in a collagenase mixture containing 1 mg/mL collagenase A, 1 mg/mL collagenase B, and 100 µg/mL type IV DNase (Sigma Aldrich). After digestion, cell pellets were separated by 30/70% Percoll, collected, and suspended in 450 µL of RPMI/5% fetal bovine serum. The isolated cells were counted and used for cytometry analysis. The leukocyte populations in blood were analyzed using 30 µL of total blood in the Trucount tubes (BD Biosciences). The leukocyte populations of CD45+ (total leukocytes), CD11b/c+ (monocyte/macrophage and neutrophil), CD8+ (cytotoxic T-cells), and CD4+ (helper T-cells) in blood and PA tissues were analyzed with the following antibody mix: CD45 BV510 (clone OX-1), CD11b/c BV650 (clone OX-42), CD8 BB515 (clone OX-8), CD4 BV711 (clone OX-35), (BD Biosciences, Le Pont-de-Claix, France).

### Evaluation of endothelial function after drug treatment

After the treatment periods, endothelial function was measured in the isolated aortic and PA rings obtained from the AIA rats within the “endothelial function” series. The vessel preparation followed the protocol described above (see section **Vascular reactivity protocol**). To determine whether the treatments improved endothelial function, rings with intact endothelium were pre-constricted with PE (10^−5^ M for PA and 10^−6^ M for aorta), and the endothelium-dependent relaxation in response to ACh (10^−11^–10^−4^ M) was recorded and compared to the AIA-vehicle group.

### Plasma cytokine measurements

The inflammatory cytokines were measured in plasma from AIA rats within the “endothelial function” series. The levels of TNF-α and IL-1β were measured using the Invitrogen™ ProcataPlex™ Mouse and Rat Mix & Match Panels kit (PPX-03-MXMFZEP, Thermo Fisher Scientific, Vienna, Austria) and then analyzed with a Luminex MAGPIX^®^ system (Luminex Corporation, Houston, TX, USA). The limit of detection was 3.66 pg/mL for TNF-α and 12.00 pg/mL for IL-1β.

### Statistical analysis

Values were expressed as the means ± SEM and were analyzed using GraphPad Prism version 9.5.1 software. Concentration-response curves to ACh in the AIA-vehicle and AIA-treated rats were compared using a two-way analysis of variance (ANOVA), followed by Bonferroni’s test. Comparisons between the two groups were made using an unpaired Student’s t-test. Variations among multiple groups were analyzed using one-way ANOVA. The relationship between the two parameters was determined by linear regression analysis, and Spearman’s correlation coefficient was calculated between these variables, with *p* < 0.05 considered statistically significant.

## Results

### Chronic DS treatment reduced arthritis severity in AIA rats

**Fig 1:**
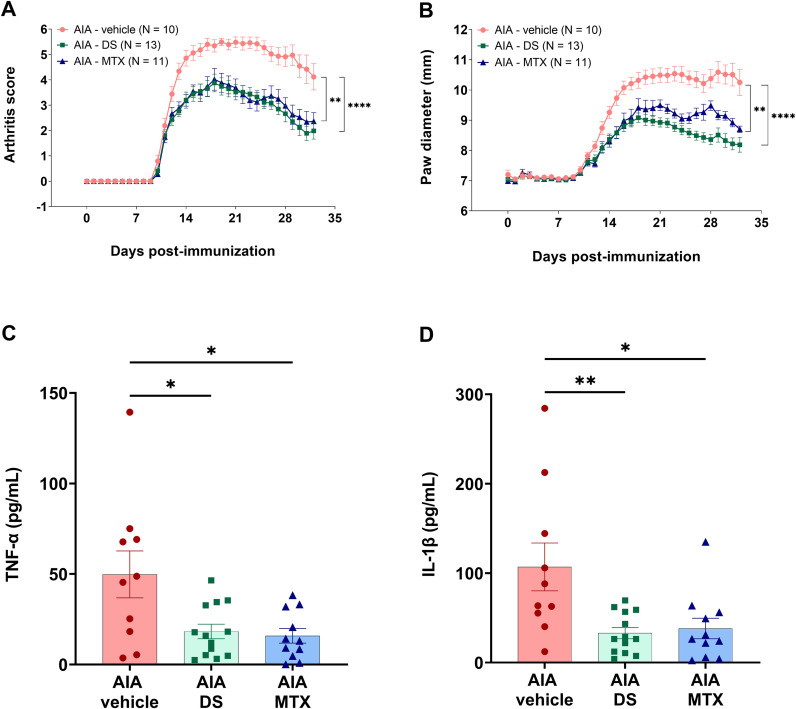
Effect of DS on arthritis inflammation in AIA rats. (**A**) arthritis scores; (**B**) paw diameter; (**C**) plasma TNF-α; and (**D**) plasma IL-1β in AIA rats treated and untreated with DS or MTX. DS (200 mg/kg/day) was orally administered, and MTX (1 mg/kg/week) was subcutaneously administered. Results are presented as the means ± SEM (N = number of rats, 10–13 rats/group). ^****^p < 0.0001, ^**^p < 0.01 and ^*^p < 0.05 vs AIA-vehicle group.

As compared to the AIA-vehicle group, arthritis score was reduced by 53% with DS treatment (*p* < 0.0001), while MTX treatment led to a 42% reduction (*p* = 0.0013) (Fig 1A). At the end of the treatment period, the arthritis scores were 2.0 ± 0.3 for the AIA-DS group and 2.4 ± 0.3 for the AIA-MTX group, compared to 4.2 ± 0.3 for the untreated group (*p* < 0.0001). Body weights were similar among the three groups (AIA-DS: 231 ± 6 g, AIA-MTX: 221 ± 4 g, AIA-vehicle: 227 ± 4 g). The Paw diameter was reduced by 18% (*p* < 0.0001) in the DS group and by 8% in the MTX group (*p* = 0.0044), Fig 1B. Additionally, compared to the AIA-vehicle group, DS treatment significantly decreased plasma levels of TNF-α (AIA-DS: 18.2 ± 4.0 pg/mL *vs* AIA-vehicle: 49.8 ± 12.9 pg/mL; *p* = 0.0160; 95% CI: 4.902–58.16) (Fig 1C) and IL-1β (AIA-DS: 32.9 ± 6.3 pg/mL *vs* AIA-vehicle: 106.9 ± 26.7 pg/mL; *p* = 0.0063; 95% CI: 18.23–129.8) (Fig 1D), S1 Table. No statistically significant differences were observed between DS and MTX treatments in terms of anti-arthritic or anti-inflammatory effects. These findings prompted further investigation into the effects of DS treatment on endothelial function.

### *Ex vivo*, direct exposure to DS induced endothelium-dependent vasorelaxation in normal rats

**Fig 2:**
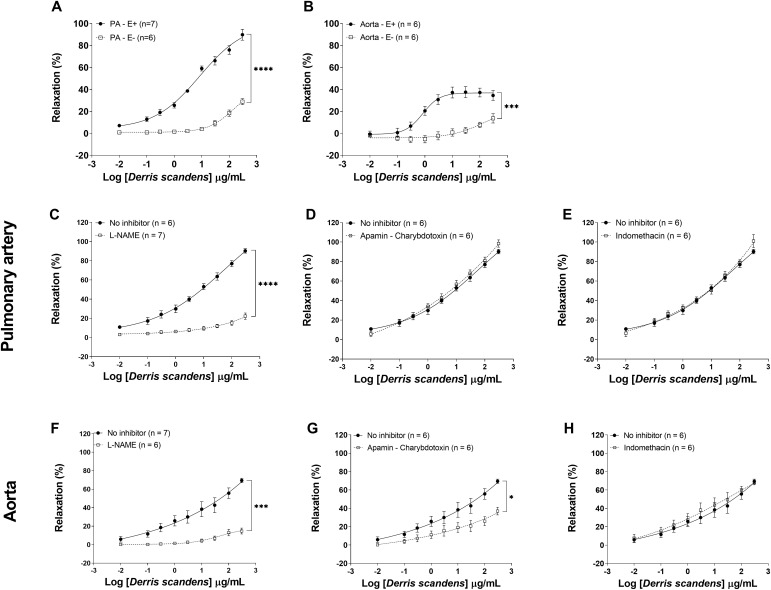
Vasorelaxant effects of DS extract and its endothelium mechanisms on the PA and aorta in normal rats. **(A-B)** Cumulative concentration-response curves of 0.01 to 300 µg/mL DS were obtained from endothelium-intact (E+) and endothelium-denuded (E-) pre-contracted with phenylephrine (PE) 10^−5^ M for **(A)** PA rings and 10^−6^ M for (**B**) aortic rings. Cumulative concentration-response curves (0.01 to 300 µg/mL) of DS were obtained from the endothelium-intact PA (**C-E**) or aorta (**F-H**) in the presence of pathway inhibitors, including (**C, F**) 10^−4^ M L-NAME (NOS inhibitor); (**D, G**) 10^−5^ M apamin plus 10^−5^ M charybdotoxin (EDHFs inhibitor); and (**E, H**) 10^−5^ M indomethacin (COX inhibitor). The relaxation responses were expressed as a percentage of PE-induced contraction. Data are expressed as means ± SEM (n = number of arterial rings, 6–7 rings/group). Two-way ANOVA of repeated measures, ^****^p < 0.0001 and ^***^p < 0.001.

Vasorelaxation was evaluated in isolated PA and aortic rings to assess the direct vascular effect of DS (Fig 2). As shown in Fig 2A and 2B, DS induced concentration-dependent vasorelaxation, with a significantly greater maximum relaxation (E_max_) observed in the PA (89.9 ± 6.5%) compared to the aorta (37.6 ± 6.1%), *p* < 0.001. In the endothelium-denuded vessels, DS-induced relaxation was markedly reduced in both PA (Fig 2A) and aorta (Fig 2B), *p* < 0.001, indicating an endothelium-dependent mechanism. In the PA rings with an intact endothelium, pre-incubation with the NOS inhibitor L-NAME significantly reduced DS-induced relaxation (Fig 2C), whereas EDHF pathway blockade (apamin plus charybdotoxin) (Fig 2D) and COX inhibition (indomethacin) (Fig 2E) had no effect. In contrast, in the aorta, DS-induced relaxation was significantly inhibited by L-NAME (Fig 2F) and apamin plus charybdotoxin (Fig 2G), but not by indomethacin (Fig 2H). These results suggest that the vasorelaxant effect of DS is mediated by the NOS pathway in the PA, while in the aorta, both NOS and EDHF pathways contribute to the effect. Consequently, the impact of DS treatment was further investigated in the context of vascular NO deficiency associated with inflammatory arthritis.

### Chronic DS treatment reduced ED in AIA rats

**Fig 3:**
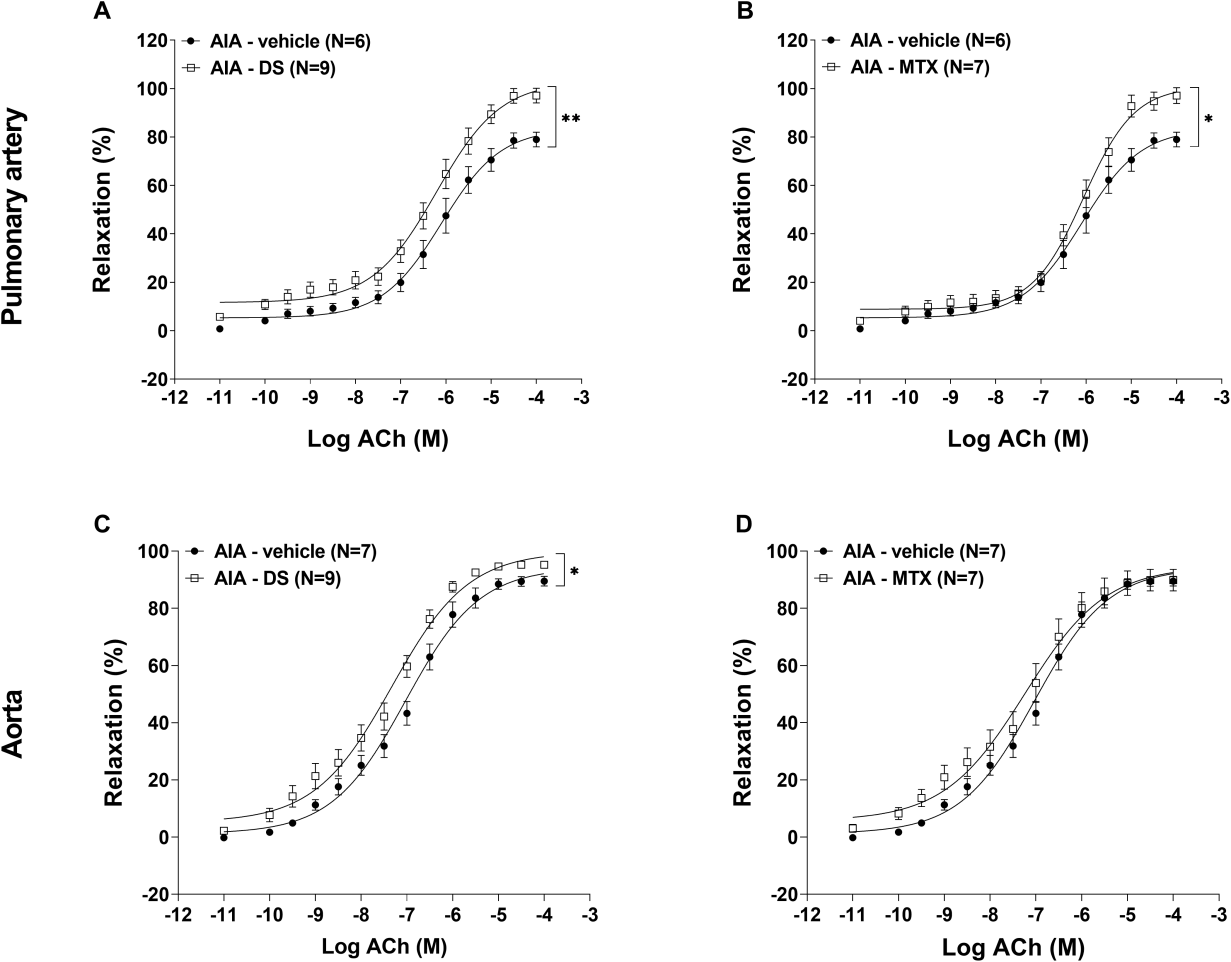
DS enhanced ACh-induced relaxation in the PA and aorta of AIA rats. Experiments were performed on the PA and aorta isolated from non-treated AIA rats and the AIA rats treated with DS and MTX. The endothelial functions of (**A, B**) the pulmonary artery (PA) and (**C, D**) the aorta were measured in endothelium-intact artery vessels pre-contracted with phenylephrine (PE), followed by cumulative concentrations of ACh 10^−11^ − 10^−4^ **M.** The relaxation effect was expressed as a percentage of PE-induced contraction. Data were expressed as means ± SEM (N = number of rats, 6–9 rats/group). Two-way ANOVA of repeated measures, **p < 0.01 and *p < 0.05 vs AIA-vehicle group.

Endothelium damage disrupts the balance between vasodilators and vasoconstrictors, contributing to the development of atherosclerosis and increased mortality in patients with RA. This study assessed the effects of chronic DS treatment on ED in two vascular beds: the aorta, representing peripheral vasculature, and the PA, due to its high expression of PDE5. In the PA, DS treatment significantly enhanced ACh-induced vasorelaxation in the AIA rats (E_max_ ACh = 97.1 ± 0.7%) compared to the AIA-vehicle group (E_max_ ACh = 79.6 ± 0.9%; *p* < 0.01) (Fig 3A). MTX treatment produced similar results (E_max_ ACh = 97.1 ± 0.9%; *p* < 0.05 vs. AIA-vehicle) (Fig 3B). In contrast, regarding the aortic response, only DS treatment (Fig 3C) significantly improved endothelial function (E_max_ ACh = 95.2 ± 0.2%) compared to the AIA-vehicle group (E_max_ ACh = 89.5 ± 0.4%; *p* < 0.05), whereas MTX treatment (Fig 3D) had no effect (E_max_ ACh = 89.9 ± 1.0%; *p* = 0.2605). Given this vascular benefit, the impact of DS treatment on cardiovascular parameters was subsequently investigated.

### Chronic DS treatment did not alter BP and ECG parameters in AIA rats

As presented in Table 1, chronic DS treatment did not alter BP or ECG parameters, except for the PR interval, which showed a slight but statistically significant increase (*p* = 0.049) compared to the AIA-vehicle group. MTX had no significant effects on any of the evaluated parameters.

**Table 1:** Effects of DS on cardiac and electrocardiographic parameters in AIA rats.

Parameters	AIA-vehicle (N = 10)	AIA-DS (N = 13)	AIA-MTX (N = 11)
HR (bpm)	262.9 ± 22.93	256.3 ± 14.87	263.9 ± 19.75
SAP (mmHg)	92.50 ± 9.49	89.00 ± 6.05	105.00 ± 8.98
DAP (mmHg)	81.98 ± 6.82	74.85 ± 8.54	85.88 ± 9.70
MAP (mmHg)	75.57 ± 8.97	84.76 ± 7.02	98.87 ± 8.47
P wave (ms)	20.05 ± 1.35	17.72 ± 1.06	17.96 ± 1.27
QRS (ms)	17.21 ± 1.59	20.36 ± 1.04	18.50 ± 0.97
PR (ms)	45.62 ± 1.95	51.23 ± 1.81^*^	49.00 ± 1.94
ST segment	51.66 ± 5.11	57.78 ± 4.43	48.66 ± 5.07
QT (ms)	69.65 ± 5.20	76.26 ± 3.73	68.86 ± 4.43
QTc (ms)	150.1 ± 12.39	157.7 ± 6.85	145.1 ± 13.16
RR (ms)	227.3 ± 17.31	242.3 ± 13.02	223.7 ± 14.11

DS treatment (200 mg/kg/day) was administered orally daily from day 11–32 post-induction, and MTX (1 mg/kg/week) was given by subcutaneous injection once a week for 3 weeks. Values are expressed as the means ± SEM (N = number of rats). Student’s unpaired t-test was used to compare two groups. ^*^p < 0.05 vs AIA-vehicle group.

### Chronic DS treatment markedly reduced vascular monocytes/macrophages and neutrophils in AIA rats

**Fig 4:**
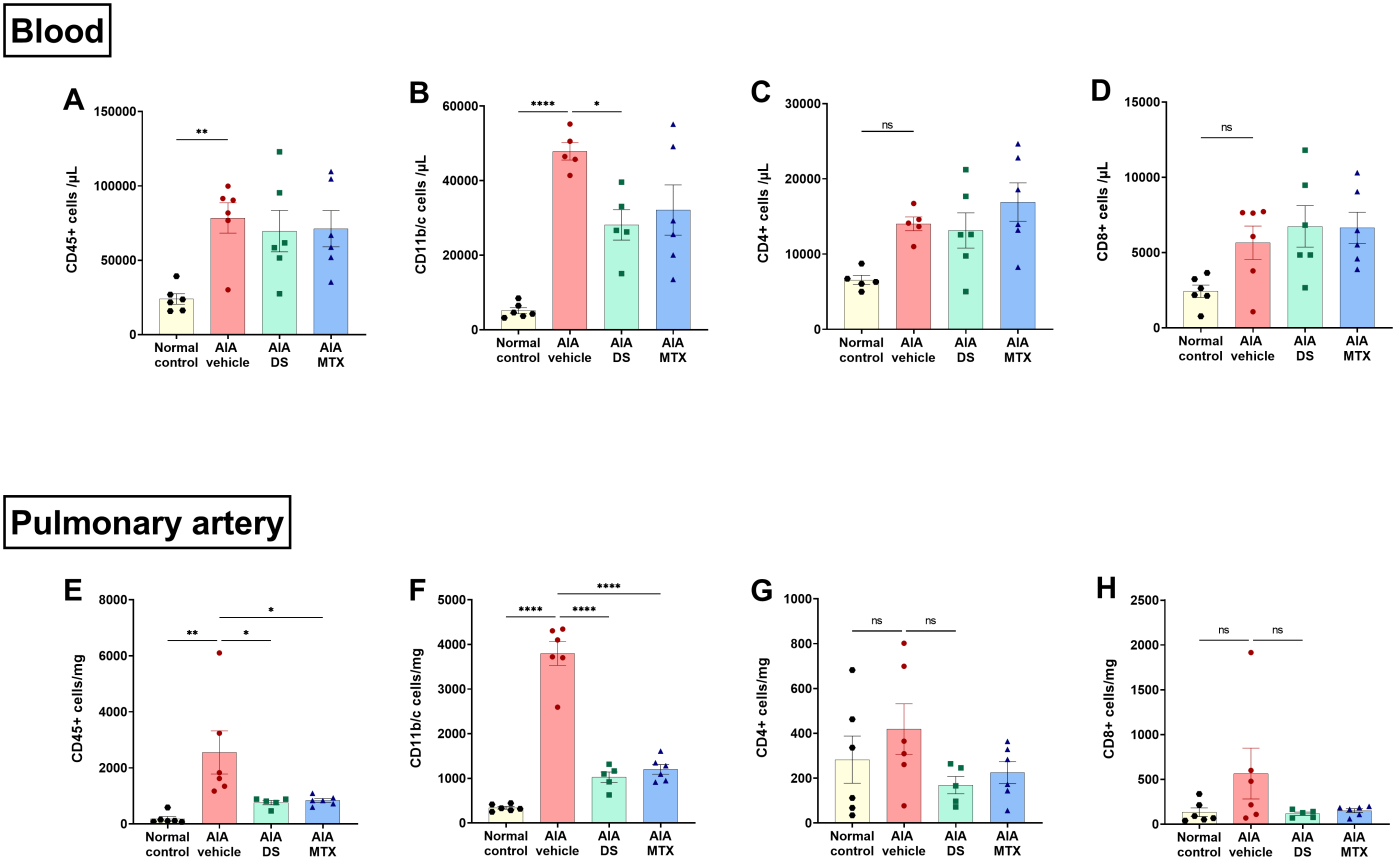
Chronic treatment with DS drastically reduced vascular monocytes/macrophages and neutrophil infiltration in AIA rats. Flow cytometry analyses of CD45 + populations were conducted on (**A-D**) blood and **(E–H)** PA samples from Non-arthritic control, AIA-vehicle, AIA-DS, and AIA-MTX rats. Various leukocyte subpopulations were measured, including (**A, E**) total leukocyte CD45+ (**B, F**) monocytes/macrophages and neutrophils CD11b/c+ cells, (**C, G**) helper T-cells CD4 + cells, and (**D, H**) cytotoxic T-cells CD8 + cells. Data for blood samples are presented as the number of stained cells per microliter (µL). Data for the PA are presented as the number of stained cells per milligram (mg) of PA. Results are expressed as means ± SEM (N = 5–6 rats/group). One-way ANOVA followed by Bonferroni’s post-test, ^****^p < 0.0001, ^**^p < 0.01, and ^*^p < 0.05. ns denotes non-significant differences.

To better understand the anti-inflammatory and vascular effects of DS, we examined its impact on immune cell populations. Flow cytometry analysis was performed to identify the leukocyte populations in the PA and peripheral blood of the AIA rats (S2 Fig). As presented in Fig 4A-4D and S3 Table, the number of total circulating leukocytes (CD45 + cells) in the AIA rats was significantly higher than that of the non-arthritic control group (*p* < 0.01). This increase was specifically driven by monocytes/macrophages and neutrophils (CD11b/c+ cells, *p* < 0.0001), while the numbers of cytotoxic T-cells (CD8 + cells) and helper T-cells (CD4 + cells) remained unchanged. Similar alterations in leukocyte populations were observed for the first time in the PA tissue of AIA rats (Fig 4E-4H and S3 Table). Following chronic DS treatment, there was a significant reduction in circulating CD11b/c+ cells (*p* < 0.0001) compared to the AIA-vehicle group. In contrast, MTX had no significant effect on peripheral leukocyte populations, likely due to high interindividual variability. In the PA tissue, the infiltration of CD11b/c+ cells was significantly reduced by both DS and MTX treatments (*p* < 0.05). These findings demonstrated for the first time that chronic DS treatment significantly mitigated systemic and vascular inflammation in AIA rats by reducing monocyte/macrophage and neutrophil populations. The next step was to investigate whether these anti-inflammatory effects were directly related to the vascular improvements observed with DS treatment.

### Correlations between leukocyte populations, endothelial function, and markers of arthritis-related inflammation

**Fig 5:**
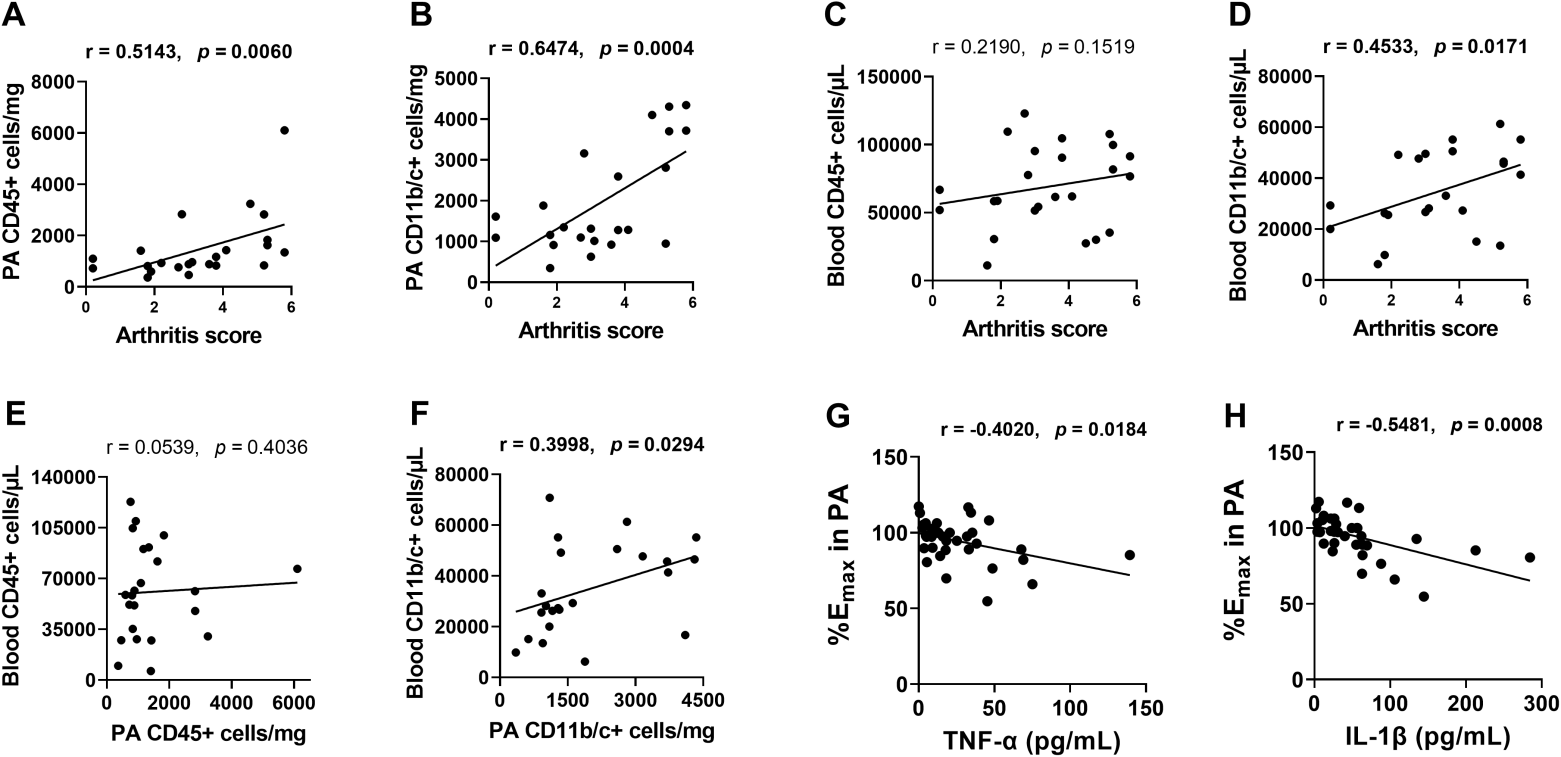
Correlations between leukocyte population, endothelial function, and markers of arthritis inflammation in AIA rats. Correlations were assessed using Spearman’s correlation coefficient in the AIA rats treated and untreated with DS or MTX. Endothelial function was quantified as the E_max_ of ACh-induced relaxation. Significant correlations (*p* < 0.05) are highlighted in bold.

Correlation analyses were performed across untreated and DS- or MTX-treated AIA rats. We observed that the number of total leukocytes (CD45+) in the PA was positively correlated with the arthritis scores; r = 0.5143, n = 23, *p* = 0.006 (Fig 5A), and the monocytes/macrophages and neutrophils (CD11b/c+) in the PA were positively correlated with the arthritis scores; r = 0.6474, n = 23, *p* = 0.0004 (Fig 5B). In circulating leukocytes, only the CD11b/c+ cell counts were significantly correlated with the arthritis scores; r = 0.4533, n = 24, *p* = 0.0171 (Fig 5C and 5D), suggesting a specific role for these cells in systemic inflammation.

We then investigated whether circulating leukocyte levels correspond to PA-infiltrated cell levels. While the CD45 + population in the PA did not correlate with circulating CD45 + counts, r = 0.0539, n = 23, *p* = 0.4036 (Fig 5E), the number of CD11b/c+ cells in the PA positively correlated with their circulating counterpart, r = 0.3998, n = 23, *p* = 0.0294 (Fig 5F). These findings suggest a possible contribution of CD11b/c+ cell reduction to the improvement in endothelial function observed with DS treatment, although this relationship warrants further investigation. Notably, PA endothelial function (as measured by ACh-induced relaxation) was negatively correlated with plasma TNF-α (r = −0.4020, n = 34, *p* = 0.0184) (Fig 5G) and IL-1β levels (r = −0.5481, n = 34, *p* = 0.0008) (Fig 5H), indicating that these cytokines may serve as biomarkers of vascular inflammation and treatment efficacy. Additionally, E_max_ values in PA were negatively correlated with the arthritis score (r = −0.4590, n = 34, *p* = 0.0063), further supporting the link between vascular improvement and arthritis severity.

### A combination of DS and MTX had no synergistic effects in AIA rats

**Fig 6:**
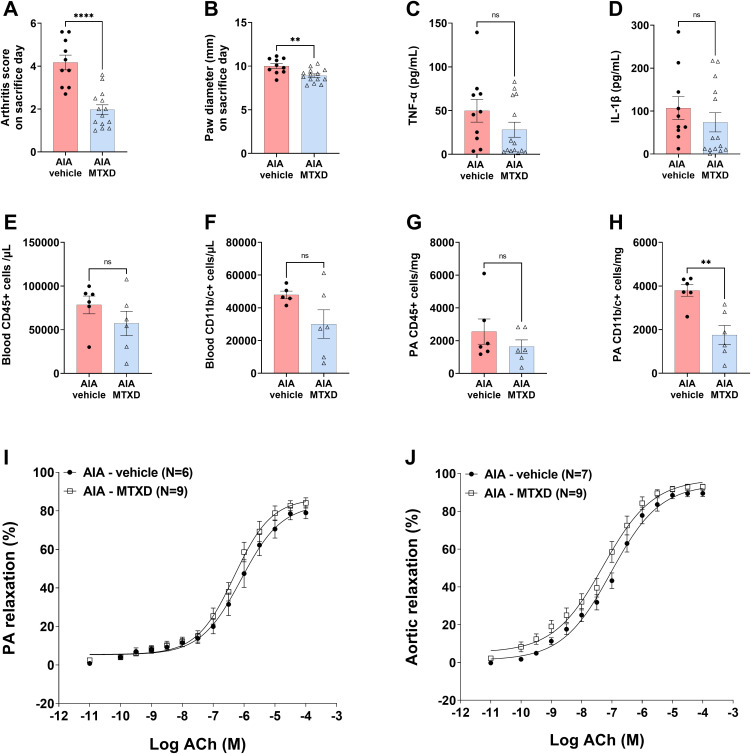
The combined effects of DS and MTX (MTXD) on arthritis inflammation and endothelial function in AIA rats. Results are expressed as means ± SEM (N = 6–14 rats/group). ^****^p < 0.0001 and ^**^p < 0.01 vs AIA-vehicle group. ns denotes non-significant differences.

Finally, the effects of the combined treatment with DS and MTX (MTXD) were evaluated. As shown in Fig 6, MTXD significantly reduced the arthritis score (Fig 6A , 1.9 ± 0.2 for the AIA-MTXD group *vs* 4.2 ± 0.3 for the AIA-vehicle group, *p* < 0.0001) and paw diameter (Fig 6B, 8.90 ± 0.20 mm for the AIA-MTXD group *vs* 10.01 ± 0.27 mm for the AIA-vehicle group, *p* = 0.0037), confirming its anti-arthritic potential. However, this combination did not confer additional benefits in reducing circulating inflammatory cytokines (Fig 6C and 6D) or total circulating (Fig 6E) or PA-infiltrated (Fig 6F) leukocyte cells. Interestingly, MTXD specifically decreased the infiltration of CD11b/c+ (monocytes/macrophages and neutrophils) in the PA (Fig 6H), suggesting a potential additive effect at the vascular level. Despite this, no improvement in endothelial function was observed in either the PA (Fig 6I) or the aorta (Fig 6J) following the combined treatment. These findings indicate that while MTXD may reduce local inflammation, it does not enhance vascular function beyond what is achieved with DS alone.

## Discussion

This study presents novel findings by demonstrating, for the first time, that chronic treatment with *Derris scandens* exerts significant anti-arthritic and vascular protective effects in the AIA rat model. These effects are primarily attributed to the plant’s potent anti-inflammatory properties, evidenced by a reduction in circulating inflammatory cytokines, specifically TNF-α and IL-1β, and a decrease in monocytes/macrophages and neutrophil populations in both the peripheral blood and pulmonary artery (PA) tissue. In addition, a direct vascular effect of *D. scandens*, through enhancement of the NO pathway, was also demonstrated.

Cardiovascular disease (CVD) remains the leading cause of mortality in patients with rheumatoid arthritis (RA), primarily due to endothelial dysfunction (ED) [28]. ED can affect both large conduit arteries, contributing to atherosclerosis, and resistance vessels, leading to organ dysfunction [6]. Systemic inflammation and vascular infiltration by leukocytes are pivotal in linking RA and ED [29]. Leukocyte recruitment and cytokine overproduction, particularly TNF-α and IL-1β, play key roles in the onset and progression of both RA and associated vascular dysfunction. During RA progression, neutrophils and other leukocytes migrate to inflamed tissues *via* endothelial adhesion and transmigration, promoting oxidative stress and vascular injury [30,31]. Although DMARDs effectively control joint inflammation, their impact on endothelial function remains controversial [6]. In recent years, there has been growing interest in phytotherapy as a complementary or adjunctive approach to RA management [32]. Some studies suggest that herbal medicines may help to reduce cardiovascular risk in RA patients, including the risk of ischemic stroke and coronary artery disease [33,34], although data specifically addressing the impact on RA-associated CVD remains limited.

In this context, we explored the pharmacological potential of *D. scandens*, a traditional anti-inflammatory plant with reported cardiovascular effects. Our results demonstrate that *D. scandens* treatment (21 days) significantly reduced arthritis severity (as measured by arthritis score and paw diameter) to a comparable extent as MTX. This supports the traditional use of *D. scandens* and highlights its potential application in systemic inflammatory arthritis, beyond its known use in musculoskeletal disorders such as low back pain and knee osteoarthritis [35]. Mechanistically, *D. scandens* treatment decreased leukocyte infiltration, particularly monocytes/macrophages and neutrophils, in both blood and PA tissue, as well as the plasma levels of TNF-α and IL-1β. These *in vivo* results are consistent with earlier *in vitro* findings, demonstrating *D. scandens*’s ability to decrease the gene expression levels of pro-inflammatory cytokines, including COX-2, iNOS, IL-6, MMP-1, and MMP-9, in non-NB-UVB HaCaT cells [20] and diminish myeloperoxidase activity and the lipoxygenase pathway [19].

While adjuvant therapies for RA have been explored extensively, few studies have addressed their impact on RA-associated CVD. Importantly, our findings show that *D. scandens* improved endothelial function in both the PA and the aorta, unlike MTX, which only improved PA responses. This vascular benefit is likely linked to the anti-inflammatory effects of *D. scandens*, as shown by the positive correlation between peripheral and vascular leukocyte counts and the negative correlation between circulating cytokine levels (TNF-α and IL-1β) and vasorelaxation. However, in our study, vascular function assessments and immune profiling were conducted in separate experimental sets, which did not allow us to establish a direct correlation between these parameters. Accordingly, further studies are warranted to clarify this causal link. Additional work is needed to elucidate the molecular mechanisms underlying *D. scandens*’s anti-inflammatory activity, such as its interaction with NF-κB signaling.

Our results also suggest a possible vascular effect of *D. scandens*, supported by its ability to induce NO-dependent vasorelaxation in healthy rats, possibly through endothelial NOS activation [36]. Despite a suspected β-adrenergic receptor antagonism of this extract [37], *D. scandens* treatment did not alter heart rate or ECG parameters, except for a slight increase in the PR interval, known to be an adverse effect of β-blockers [38]. This stable heart rate may be attributed to the fact that this parameter was not elevated under arthritic conditions.

Although some studies suggest that combining herbal treatment with DMARDs can reduce cardiovascular risk [33,34,39], our data show that co-administration of *D. scandens* and MTX, while effective in alleviating arthritis symptoms, abolishes the vascular benefits observed with *D. scandens* alone in AIA rats. This lack of synergy, or even antagonism, may result from pharmacokinetic interactions. Further studies should investigate this possibility, with particular attention to cytochrome P450 enzyme activity [40] and membrane transporters, which are known to modulate MTX distribution and efficacy [41]. Otherwise, improved stability and delivery of methotrexate may reduce its interaction with other treatments. The use of methotrexate-loaded biodegradable nanoparticles may constitute an innovative strategy [42].

This study has some limitations. All experiments were conducted in male rats, and the results must be confirmed in female rats to evaluate potential sex differences. Additionally, our focus was on the pharmacological evaluation of the *D. scandens* extract rather than on identifying its active constituents. Future phytochemical studies should aim to characterize the bioactive compounds responsible for the observed effects.

## Conclusion

While phytotherapies for rheumatoid arthritis (RA) have been extensively explored, there is a notable scarcity of studies examining their effects on RA-associated cardiovascular disorders. This study provides evidence that *D. scandens* is a promising adjuvant therapeutic candidate for alleviating endothelial dysfunction and reducing leukocyte infiltration and pro-inflammatory cytokine levels in rats with adjuvant-induced arthritis. These findings emphasize the traditional use of *D. scandens* as an anti-inflammatory plant. Given its vascular and anti-arthritic benefits, *D. scandens* merits further investigation as a potential alternative or adjunct therapy in clinical settings for managing RA and its cardiovascular comorbidities.

## Supporting information

S1 Table Plasma levels of cytokines in AIA rats. (PDF)

S2 Fig Gating strategies. (PDF)

S3 Table Leukocyte populations. (PDF)
