# Supplementary material for: Anti-arthritic and endothelial protective effects of Derris scandens extract in adjuvant-induced arthritis in rats
Source: PLoS One. 2025 Dec 11;20(12):e0337472. doi: 10.1371/journal.pone.0337472 (PMC12697982; doi:10.1371/journal.pone.0337472)
Supplement: S1 Table — (PDF) [file pone.0337472.s001.pdf]

**S1 Table. Plasma levels of cytokines in AIA rats.** AIA rats were treated with either vehicle (AIA-vehicle), propylene glycol, 200 mg/kg/day (p.o.) *Derris scandens* ethanolic stem extract (AIA-DS), 1 mg/kg (s.c.) methotrexate once a week (AIA-MTX), or a combination of *Derris scandens* ethanolic stem extract at 200 mg/kg/day (p.o.) plus methotrexate at 1 mg/kg (s.c.) once a week (AIA-MTXD).

| **Group** | **TNF-α (pg/mL)** | **IL-1β (pg/mL)** |
|---|---|---|
| AIA-vehicle | 49.77 ± 12.94 | 106.9 ± 26.73 |
| AIA-DS | 18.23 ± 4.03$^{*}$ | 32.93 ± 6.29$^{**}$ |
| AIA-MTX | 15.80 ± 4.04$^{*}$ | 38.08 ± 11.51$^{*}$ |
| AIA-MTXD | 28.09 ± 8.59 | 73.79 ± 22.39 |

Values are presented as mean ± SEM (N = 10 – 13, N = Number of rats).

$^{**}p<0.01$ and $^{*}p<0.05$ vs AIA-vehicle group.
