# Supplementary material for: Anti-arthritic and endothelial protective effects of Derris scandens extract in adjuvant-induced arthritis in rats
Source: PLoS One. 2025 Dec 11;20(12):e0337472. doi: 10.1371/journal.pone.0337472 (PMC12697982; doi:10.1371/journal.pone.0337472)
Supplement: S2 Fig — (PDF) [file pone.0337472.s002.pdf]

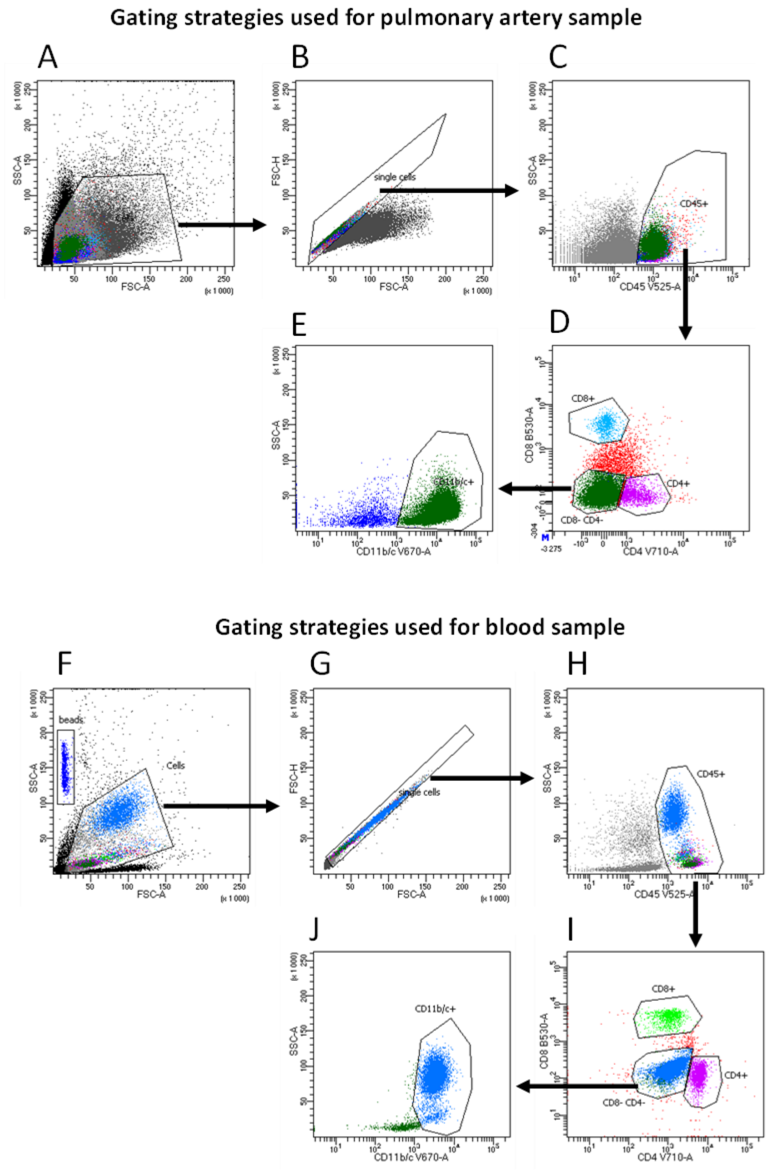

S2 Fig. Gating strategies. Gating strategies used to analyze leukocyte subsets in pulmonary artery and blood samples. **A–E** show representative analyses from pulmonary artery samples, and **F–J** from blood samples, including total leukocytes, granulocytes, monocytes/macrophages, total T cells, CD4$^+$ T cells, and CD8$^+$ T cells. Cells were first identified based on size and granularity (**A and F**; FSC vs SSC), followed by single-cell selection **(B and G)**. Total leukocytes were then identified by expression of the pan-leukocyte marker CD45 **(C and H)**. CD4$^+$ and CD8$^+$ T cells were identified within the CD45$^+$ population based on CD4 and CD8 expression **(D and I)**. Among CD4$^-$CD8$^-$ cells, monocytes, granulocytes, and macrophages were distinguished by CD11b/c expression **(E and J)**. Cell quantification in blood samples was performed using the TruCount method. Counting beads was identified using the FSC vs. SSC gate **(F)**.
