# Supplementary material for: Anti-arthritic and endothelial protective effects of Derris scandens extract in adjuvant-induced arthritis in rats
Source: PLoS One. 2025 Dec 11;20(12):e0337472. doi: 10.1371/journal.pone.0337472 (PMC12697982; doi:10.1371/journal.pone.0337472)
Supplement: S3 Table — (PDF) [file pone.0337472.s003.pdf]

S3 Table. Leukocyte populations. Leukocyte populations in blood (cells/mL) and pulmonary artery tissue (cells/mg) of AIA rats. AIA rats were treated with either vehicle (AIA-vehicle), propylene glycol, 200 mg/kg/day (p.o.) *Derris scandens* ethanolic stem extract (AIA-DS), 1 mg/kg (s.c.) methotrexate once a week (AIA-MTX), or a combination of *Derris scandens* ethanolic stem extract at 200 mg/kg/day (p.o.) plus methotrexate at 1 mg/kg (s.c.) once a week (AIA-MTXD).

Group	CD45+	CD11b/c+	CD4+	CD8+
**Blood (cells/mL)**				
Normal control	23918.2 ± 1441.2	5104.0 ± 808.8	6563.2 ± 274.2	2428.0 ± 168.1
AIA-vehicle	78384.8 ± 4163.0**	47868.2 ± 2336.0****	14021.8 ± 411.18	5649.7 ± 452.1
AIA-DS	69534.0 ± 5673.6$^{\#}$	28136.0 ± 4075.0	13137.7 ± 954.3	6736.2 ± 562.5
AIA-MTX	71179.5 ± 4960.8	32114.3 ± 2751.9	16910.8 ± 1071.2	6634.5 ± 423.6
AIA-MTXD	57232.7 ± 5696.9	30079.3 ± 3555.0	11413.7 ± 1075.1	5445.5 ± 501.8
**Pulmonary artery tissue (cells/mg)**				
Normal control	184.0 ± 33.1	340.3 ± 12.5	282.5 ± 43.0	133.4 ± 19.7
AIA-vehicle	2551.4 ± 314.7**	3793.9 ± 108.4****	418.9 ±46.1	564.7 ± 115.7
AIA-DS	761.9 ± 34.8$^{\#}$	1025.3 ± 52.6$^{\#\#\#\#}$	169.2 ± 17.2	116.3 ± 8.4
AIA-MTX	835.3 ± 28.4$^{\#}$	1199.8 ± 44.3$^{\#\#\#\#}$	224.8 ± 19.9	150.4 ± 9.1
AIA-MTXD	1637.3 ± 166.7	1750.4 ± 180.2	273.5 ±27.4	169.9 ± 16.5

Values are presented as mean ± SEM (N = 5 – 6, N = Number of rats).

$^{****}p<0.0001$ and $^{**}p<0.01$ vs Normal control.

$^{\#\#\#\#}p<0.0001$ and $^{\#}p<0.05$ vs AIA-vehicle group.
